# Companion diagnostics and predictive biomarkers for PD-1/PD-L1 immune checkpoint inhibitors therapy in malignant melanoma

**DOI:** 10.3389/fimmu.2024.1454720

**Published:** 2024-10-28

**Authors:** Zeping Wang, Xiaojing Zou, Haiyan Wang, Zhihui Hao, Gebin Li, Shuaiyu Wang

**Affiliations:** ^1^ College of Veterinary Medicine, China Agricultural University, Beijing, China; ^2^ Beijing Biomedical Science and Technology Center, Zhaofenghua Biotechnology (Nanjing) Company Limited, Beijing, China; ^3^ Key Biology Laboratory of Chinese Veterinary Medicine, Ministry of Agriculture and Rural Affairs, Beijing, China; ^4^ National Center of Technology Innovation for Medicinal Function of Food, National Food and Strategic Reserves Administration, Beijing, China

**Keywords:** PD-1, PD-L1, biomarkers, CDx, immunotherapy

## Abstract

Programmed cell death receptor 1 (PD-1), when bound to the ligand programmed death-ligand 1 (PD-L1), can suppress cellular immunity and play a critical role in the initiation and development of cancer. Immune drugs targeting these two sites have been developed for different cancers, including malignant melanoma. The accompanying diagnostic method has been approved by the FDA to guide patient medication. However, the method of immunohistochemical staining, which varies widely due to the antibody and staining cut-off values, has certain limitations in application and does not benefit all patients. Increasing researches begin to focus on new biomarkers to improve objective response rates and survival in cancer patients. In this article, we enumerated three major groups, including tumour microenvironment, peripheral circulation, and gene mutation, which covered the current main research directions. In the future, we hope those biomarkers may be used to guide the treatment of patients with malignant melanoma.

## 1 Introduction

As aging population keeps growing, deaths and disability-adjusted life years (DALYs) due to non-communicable diseases (NCDs) are ticking up. According to the latest report from the World Health Organization (WHO), the number of people killed by NCDs in 2019 rose by 28% as compared to 2000, with cancer being the second cause of death (9.3 million) (1). Melanoma is caused by malignant transformation of melanocytes, with a high incidence across the world. It is the fifth, sixth most common cancer in men, women respectively (2). Before the advent of cancer immunotherapy, metastatic melanoma (MM) had a poor survival prognosis. Nowadays, there are several immunotherapy options for MM, such as anti-PD-1 and anti-cytotoxic T-lymphocyte-associated protein 4 (CTLA4) antibody therapy, which represent desirable efficacy (3, 4).

Programmed cell death protein 1(PD-1) is an important immunosuppressive apoptotic process. It regulates the immune system and promotes self-tolerance by down-regulating the immune system’s response to the body’s cells and by suppressing T cell activity. Programmed cell death 1 ligand 1 (PD-L1) is often overexpressed in tumour cells and is an apoptotic process to bind to PD-1 *in vivo*, signaling immunosuppression and reducing T cell proliferation and activation, which suggests that the PD-1/PD-L1 pathway is a mechanism of tumour immune escape (5, 6). PD-L1 expression is upregulated on tumour cells or tumour microenvironments such as tumour-infiltrating lymphocyte, and decreases T cell activity by binding to PD-1 on tumour antigen-specific T cell (5, 7, 8). Blocking the PD-1/PD-L1 pathway with anti-PD-1 or anti-PD-L1 antibodies can restore multiple effector functions of T cell (9), which in turn can alleviate cancer progression (10).

Despite the promising potential of immunotherapy, only a small proportion of patients achieve durable responses to monotherapy, and therefore predictive biomarkers are used for the prognosis of immunotherapy (11). Companion diagnostic (CDx) is an *in vitro* diagnostic technique associated with targeted drugs, which selects the most suitable drug groups and target therapy by measuring the expression levels of biomarkers in the body. The Food and Drug Administration (FDA) has approved several CDx methods for PD-1 immunotherapy, such as the use of PD-L1 22C3 monoclonal antibody for immunohistochemical staining of tumour tissue from patients with non-small-cell lung carcinoma (NSCLC). Patients with Tumour Proportion Score (TPS) higher than 50% can be treated with targeted immunotherapy with PD-1 monoclonal antibody. With the advancement of the research, the CDx method of detecting PD-L1 expression in tumour tissue is not of the greatest benefit to patients. Because of differences in sampling sites, some PD-L1-negative patients may still benefit from PD-1 immunotherapy (12). To date, many patients benefit greatly from guided therapy with CDx, but we are still looking forward to the discovery of additional diagnostic methods and biomarkers to improve the objective response rate (ORR) of patients to immunotherapy. There are several biomarkers associated with the response to anti-PD-1/anti-PD-L1 therapy including T cell infiltration, mutant genome, circulating extracellular vesicles, and circulating soluble PD-L1 expression, etc. In addition, we expect RT-PCR to replace IHC as a CDx method for cancer patients.

This review will summarize the CDx approaches and prospective biomarkers associated with PD-1/PD-L1 immunotherapy for malignant melanoma, providing the latest advances in melanoma immunotherapy and new strategies for future development of cancer therapy.

## 2 PD-1/PD-L1 immunotherapy and malignant melanoma

Activation of the PD-1/PD-L1 signalling pathway can suppress cellular immunity in cancer patients, thus maintaining tumour immune tolerance. Modern research has shown that T cell activation depends primarily on dual signals, with the first being major histocompatibility complex (MHC) antigen presentation that binds to the T-cell receptor (TCR), and the second signal consisting of a costimulatory signal and a coinhibitory signal (13).

**Figure 1:**
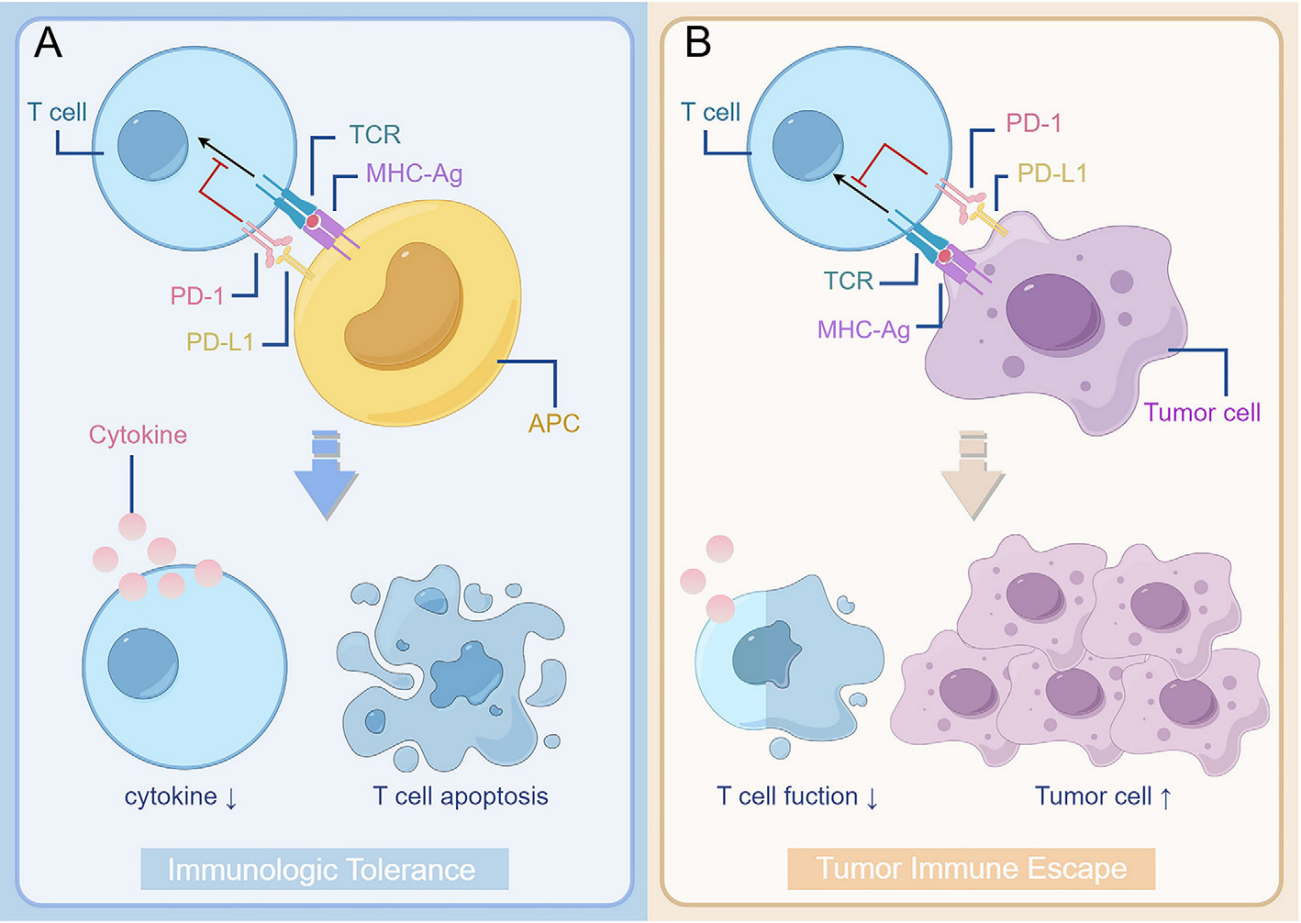
The interaction between PD-1 and PD-L1 reduces T cell function and plays a role in promoting the development of tumour. **(A)** Binding of PD-1 on the surface of T cells to PD-L1 on the surface of antigen-presenting cells results in suppression of T cell function, such as reduced cytokine secretion and apoptosis. It forms immunologic tolerance and reduces ineffective or harmful immune responses in normal organisms. **(B)** However, as binding of PD-1 on the surface of T cell to PD-L1 on the surface of tumour cells takes place in the tumour patient body, it can cause the tumour cell’s immunity escape. The decrease of T cell function leads to the proliferation of tumour cells. TCR, T cell receptor; MHC, major histocompatibility complex; APC, antigen-presenting cells; PD-1, programmed cell death protein 1; PD-L1, programmed cell death 1 ligand 1.

The combination of PD-1 expressed on T cell surface and PD-L1 expressed on tumour cell or antigen presenting cell surface can effectively inhibit T cell activation, reduce cytokine production and even cause T cell death (**Figure 1**). Activation of the PD-1/PD-L1 signalling pathway in normal organisms is primarily to reduce ineffective or deleterious immune responses and maintain immune tolerance. However, its negative effects on immunity can lead to immune escape by tumour cells (14), which is known as the “adaptive immune mechanism” of tumour cells (15). Tumour cell expressed PD-L1 binds to its receptor to act as a pro-cancer factor, activating proliferation and survival signalling pathways (16).

As the PD-1/PD-L1 pathway mediated immunosuppression is reversible, blocking PD-1/PD-L1 signalling therapy represents a breakthrough in tumour immunotherapy. This technique has been used in preclinical trials and clinical stages for the treatment of various malignant tumours. FDA approved the use of PD-1/PD-L1 immunotherapies for patients with malignant melanoma, including pembrolizumab, nivolumab, and atezolizumab, without specific CDx regimen for melanoma patients. There are enough clinical data to demonstrate the efficacy of PD-1/PD-L1 immunotherapy in patients with melanoma.

Pembrolizumab is the first monoclonal antibody anti-PD-1 drug FDA-approved and is generally used in patients with advanced melanoma or as an adjuvant therapy after surgery. In a Phase I trial (KEYNOTE-001), pembrolizumab had an antitumour effect in patients with locally advanced or MM (17). A randomized, controlled, phase III trial (KEYNOTE-006) revealed that pembrolizumab prolonged progression-free survival (PFS) and overall survival (OS) in patients with advanced melanoma as compared with the monoclonal antibody anti-CTLA-4 drug ipilimumab, and the incidence of high-grade adverse reactions was low (18). These trials demonstrated durable benefits with pembrolizumab in the majority of patients, with one study evaluating pembrolizumab’s durable benefits for the KEYNOTE-001 and KEYNOTE-006 trials and assessment of stable disease (SD) at week 12 or week 24. As for melanoma patients with partial response (PR) or complete response (CR), the earlier the CR outcome, the better the prognosis (19). This indicates the need for regular assessment of patient response during treatment to predict subsequent treatment progress. Patients with completely resected stage III melanoma were randomly assigned to pembrolizumab (505 patients) or placebo (3 patients) for 1 year, and relapse free survival and drug safety were assessed. The results showed that pembrolizumab, as an adjunct therapy after surgery for high-risk stage III melanoma, offered more benefits to patients than placebo, without new toxic side effects (20).

Nivolumab, a human-derived IgG4, was found to have excellent anti-solid tumour activity and safety in a phase I trial (21). During 18 months of follow-up, nivolumab showed a higher 12-month recurrence-free survival rate (70.5%: 60.8%) and a lower rate of adverse events (3.4%: 14.4%) than ipilimumab (22). In a phase III, randomized, double-blind trial (Checkmate 066) of patients with untreated MM without v-raf murine sarcoma viral oncogene homolog B1 (BRAF) mutations, nivolumab was found to have a much higher ORR and OS than dacarbazine, and the incidence of adverse events was lower (23).

Atezolizumab, an anti-PD-L1 monoclonal antibody, was first approved as a monotherapy or combination therapy for patients with urothelial, lung, or breast cancer. In a phase I trial, atezolizumab demonstrated desirable anti-metastatic melanoma efficacy (ORR = 30.2%) and a long duration of response, with a median response period of up to 62 months (24). atezolizumab is often used as a member of adjuvant or combination therapy, and in a phase I study the combination safety and clinical activity of the MEK inhibitor cobimetinib with the anti-PD-L1 antibody atezolizumab was available regardless of Kirsten rat sarcoma viral oncogene homologue (KRAS)/BRAF status (25). A phase III trial showed that the combination of cobimetinib-vemurafenib with atezolizumab was more effective than placebo in BRAFV600 mutation-positive unresectable or advanced melanoma with no greater risk of adverse effects (26).

Pembrolizumab or nivolumab is often used in combination with CTLA-4 immune checkpoint inhibitors, and its clinical benefit in patients with metastatic or advanced melanoma has been noted in numerous literature. In a phase II clinical trial (Checkmate 069), 142 patients with untreated advanced melanoma were randomly assigned in a 2:1 ratio to compare the efficacy of nivolumab plus ipilimumab combination with that of ipilimumab monotherapy; clinical ORR improved significantly in patients receiving combination therapy, but the incidence of adverse events increased (27). nivolumab demonstrated desirable clinical response to combination and monotherapy in the phase III clinical trial (Checkmate 067), where patients were followed for an additional 6.5 years, and the longest progression-free survival and median OS were found to be 27.6 months and 72.1 months in the combination group, respectively (28). It was precisely because of the increased incidence of adverse events with combination therapy that patients with cancer were generally treated with PD-1/PD-L1 monotherapy, followed by ipilimumab as second-line therapy when treatment failed. One study recruited patients with melanoma who had failed PD-1/PD-L1 immunotherapy to pembrolizumab in combination with low-dose ipilimumab, with an ORR of 29%, a median survival time (MST) of 16.6 months, and a 3% rate of adverse events (29). Whether it is monotherapy, sequential administration, or combination therapy, the recommendations of oncologists must be strictly followed in order to achieve greater clinical benefits for patients.

## 3 Companion diagnostics of PD-1/PD-L1

CDx is an *in vitro* Companion Diagnostic Devices associated with targeted drugs, and the FDA issued guidelines for CDx in 2014 to help screen specific groups of patients who could benefit from the treatment product, to improve the prognosis of treatment and reduce medical expenditure (30). There is substantial clinical evidence that the higher the level of PD-L1 expression by tumour cells or tumour-infiltrating lymphocyte, the higher the clinical response rate for patients using PD-1/PD-L1 immunotherapy. It is suggested that PD-L1 CDx plays an important role in guiding the use of antibody in tumour immunotherapy, improving the accuracy of tumour pathological detection and prognostic assessment, and the development of PD-L1 CDx reagents is the inevitable trend of immune checkpoint targeted therapy in the future. FDA-approved CDx methods for PD-1/PD-L1 immunotherapy are listed in **Table 1**.

**Table 1:** FDA approvals of companion diagnostic assays for PD-1/PD-L1 immunotherapy.

Drug	Diagnostic name	Manufacturer	Biomarkers	Grant date and indication sample type
**Keytrud** **(Pembrolizumab)**	PD-L1 IHC 22C3 pharmDx	Dako	PD-L1 protein expression (Any staining intensity, TPS <50% or ≥50%)	2015, NSCLC 2018, Cervical Cancer 2019, HNSCC 2019, ESCC 2020, TNBC
	FoundationOne CDx	Foundation Medicine	TMB ≥10 mutations per megabase	2020, Solid Tumours
**Libtayo** **(Cemiplimab-rwlc)**	PD-L1 IHC 22C3 pharmDx	Dako	PD-L1 protein expression (TPS ≥50%)	2021, NSCLC
**Opdivo (Nivolumab) in combination with Yervoy (Ipilimumab)**	PD-L1 IHC 28-8 pharmDx	Dako	PD-L1 protein expression (Tumour cell staining ≥1%)	2020, NSCLC
**Tecentriq** **(Atezolizumab)**	Ventana PD-L1 (SP142) Assay	Ventana	PD-L1 protein expression (IC ≥5%) PD-L1 protein expression (TC ≥50 or IC ≥10%)	2016, Urothelial Carcinoma 2018, NSCLC
	Ventana PD-L1 (SP263) Assay		PD-L1 protein expression	2021, NSCLC

PD-L1, programmed cell death 1 ligand 1; IHC, immunohistochemistry; CDx, companion diagnostic; TPS, Tumour Proportion Score; TMB, tumour mutation burden; IC, immune cells; TC, tumour cells; NSCLC, non-small cell lung cancer; HNSCC, head and neck squamous cell carcinoma; ESCC, esophageal squamous cell carcinoma; TNBC, triple-negative breast cancer. Information was collected from the “List of Cleared or Approved Companion Diagnostic Devices (*in vitro* and Imaging Tools)” (31).

Immunohistochemistry (IHC) is an effective and commonly used method to evaluate the expression of PD-L1 in tumour tissues. It is widely used in many kinds of tumours, including NSCLC, MM and so on, to identify or aid in the prediction of patients who may benefit from immunotherapy. The principle is to use antigen-antibody specific binding, through chemical reaction to make the color reagent labeled antibody to determine the tissue-cell antigen, its location, qualitative and relative quantitative study. Currently, the FDA has approved tests for PD-1/PD-L1 concomitant diagnostic with anti-PD-L1 monoclonal antibody 22C3,28-8, SP142, and SP263 to guide clinical use of pembrolizumab, nivolumab, and atezolizumab, respectively.

In a phase I study of pembrolizumab in patients with advanced NSCLC, the ORR was 19.4%. However, when patients were grouped using the concomitant diagnostic, the ORR with pembrolizumab increased to 45.2% in patients with TPS greater than 50% (32). In another study of clinical efficacy of pembrolizumab in combination with a CDx in patients with NSCLC, the ORR was 52% was found for patients with TPS greater than 50% (33). Similar results were seen in patients with PD-L1-positive melanoma, where ORR, MST, and OS were significantly higher than in patients with PD-L1-negative melanoma (34).

Although the advent of CDx benefits numerous patients, IHC still has many limitations: the IHC protocol for PD-L1 detection varies greatly due to the difference of antibody and staining threshold (35); the interchangeable detection method and PD-L1 positive cut-off criterion may lead to the discordance of PD-L1 status classification in some patients; the limitations of the current IHC methods for assessing PD-L1 expression in terms of reproducibility and sampling variability are illustrated (36). Current detection of PD-L1 focuses on biopsy samples, which are not representative of the entire tumour, and studies have found that in a substantial proportion of patients, the PD-L1 positive rate in biopsy samples is lower than that in surgical resection samples, leading to false-negative results and patients misdiagnosis (37). Moreover, IHC results need to be evaluated by a pathologist, which is subjective. In a study that used multiple PD-L1 IHC to detect tumour specimens, 22C3 was the most sensitive monoclonal antibody, followed by 28-8 and finally SP-142. This shows the problem that if the physician selects only one IHC test, the PD-L1 status of the patient’s tumour sample in the results may not be accurate. This may be related to the differences in the number, size and accessibility of specific epitopes recognized by different antibodies (38).

In view of the limitations of PD-L1 IHC detection, we need a more convenient and accurate CDx method. As research advances, molecular diagnosis will become the fastest growing and most profitable field, including real-time PCR, *in situ* hybridization and new generation sequencing technology. The expression level of PD-L1 mRNA in peripheral blood of patients with gastrointestinal cancer by qRT-PCR was compared with that by PD-L1 IHC in paired tumour tissues, but it was not statistically significant. However, it was found that gastric cancer patients with high expression of PD-L1 in blood had better response to immunotherapy (35). Similar trials were conducted in patients with non-metastatic renal clear cell carcinoma and patients with primary NSCLC, both of which used tumour tissue from patients to compare the concordance of the qRT-PCR method with the IHC method. The results showed high consistency between the two methods, but they were also related to the selection of anti-PD-L1 monoclonal antibody (39, 40). Compared with IHC, qRT-PCR is a simple, accurate, easy-to-use, time-and cost-saving, semi-quantitative method that does not depend on the evaluation of observers and is expected to be a new CDx method for melanoma patients (41).

## 4 Prospective biomarkers for PD-1/PD-L1 immunotherapy

**Figure 2:**
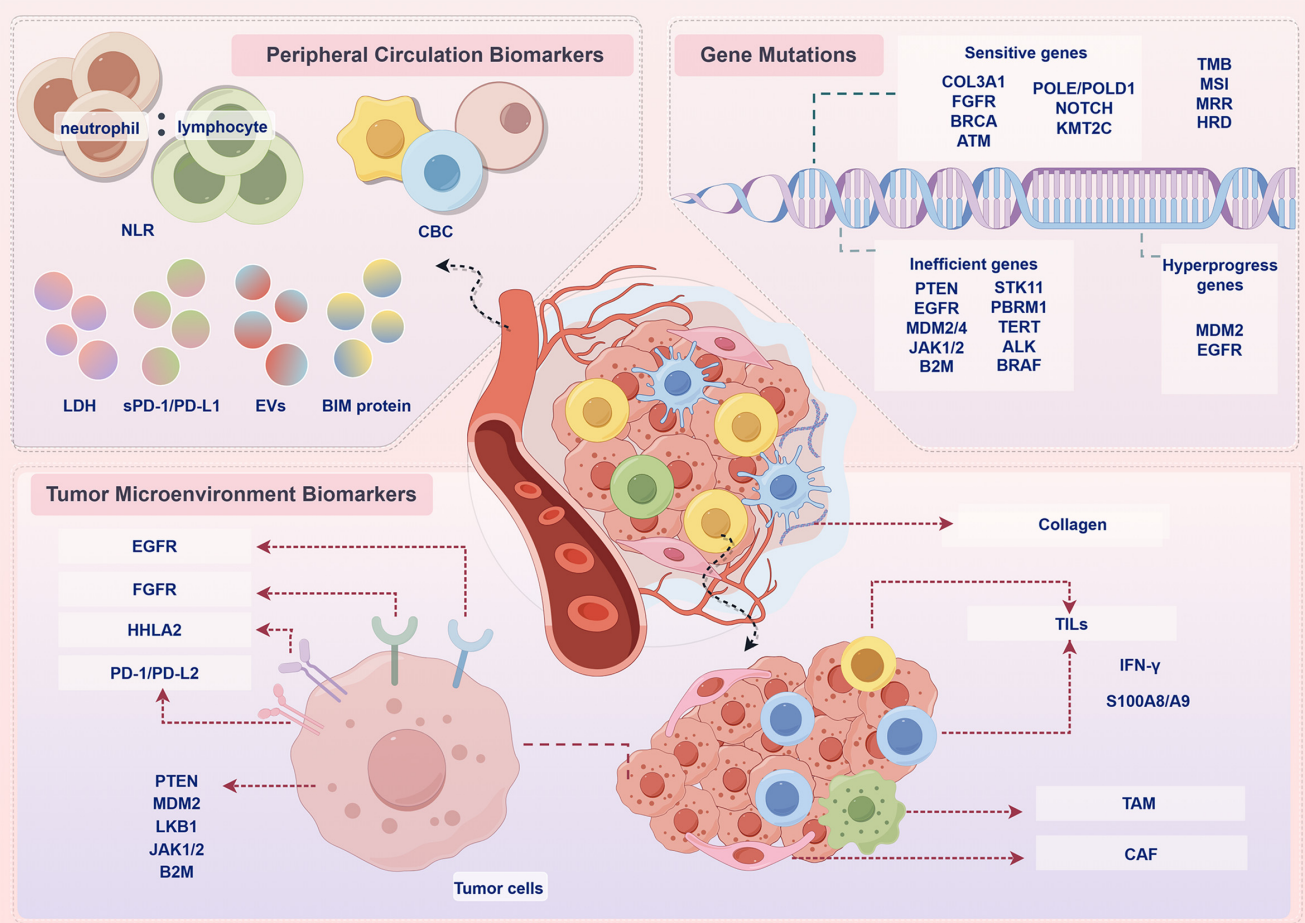
Prospective biomarkers for PD-1/PD-L1 immunotherapy. In addition to PD-L1 expression status, the biomarkers were classified into peripheral circulation biomarkers, tumour microenvironment biomarkers, gene mutations according to their characteristics. NLR, neutrophil-to-lymphocyte ratio; CBC, complete blood count; LDH, lactate dehydrogenase; EVs, extracellular vesicles; BIM, BCL-2 interacting mediator of cell death; COL3A1, collagen alpha-1(III) gene; FGFR, fibroblast growth factor receptor; BRCA, breast cancer susceptibility gene; ATM, ataxia telangiectasia-mutated gene; POLE, polymerase epsilon; POLD, DNA polymerase delta; PTEN, phosphatase and tensin homolog deleted on chromosome 10; EGFR, epidermal growth factor receptor; MDM2, mouse double minute 2 homolog; JAK, Janus kinase; B2M, β2-microglobulin; STK11, serine/threonine kinase 11; PBRM1, polybromo 1; TERT, telomerase reverse transcriptase; ALK, anaplastic lymphoma kinase; BRAF, v-raf murine sarcoma viral oncogene homolog B1; TMB, tumour mutational burden; MSI, microsatellite instability; MRR, DNA mismatch repair; HRD, homologous recombination deficiency; HHLA2, human endogenous retrovirus-H long repeat-associating 2; PD-L2, programmed cell death 1 ligand 2; LKB1 liver kinase B1; TILs, tumor-infiltrating lymphocytes; TAM, tumour-associated macrophage; CAFs, cancer-associated fibroblast; IFN-γ, interferon-γ.

Currently, PD-L1 levels in tumour tissue are a commonly used marker to predict the efficacy of PD-1/PD-L1 immunotherapy. However, studies have shown that, no matter the patient’s tumour tissue expresses PD-L1 or not, they can equally benefit from immunotherapy. This illustrates the importance of finding novel and effective biomarkers that predict the efficacy of PD-1/PD-L1 inhibitors, as well as the need for innovation in CDx assays (42). In addition to PD-L1 expression status, the biomarkers were classified into tumour microenvironment biomarkers, peripheral circulation biomarkers and gene mutations according to their characteristics (**Figure 2**), as discussed below.

### 4.1 Tumour microenvironment biomarkers

The internal environment in which tumour cells are produced and live is called the tumour microenvironment (TME), including haematopoietic cells, fibroblasts, blood and lymphatic endothelial cells, as well as the surrounding extracellular matrix and soluble factors they produce. It is characterized by hypoxia, chronic inflammation and immunosuppression, which forms a very complex network of mechanisms. It plays an important role in the development of tumour. Biomarkers associated with PD-1/PD-L1 immunotherapy include PD-L2, human endogenous retrovirus-H long repeat-associating 2 (HHLA2), CD8+ T cells, B-cell receptor (BCR), tumour-associated macrophage (TAM), collagen alpha-1 (III) (COL3A1), phosphatase and tensin homolog deleted on chromosome 10 (PTEN), epidermal growth factor receptor (EGFR), mouse double minute 2 homolog (MDM2), fibroblast growth factor receptor (FGFR), serine/threonine kinase 11 (STK11), interferon-γ (IFN-γ), Janus kinases 1/2 (JAK1/2), β2-microglobulin (B2M), cancer-associated fibroblasts (CAFs), S100A8/A9 and so on (**Table 2**).

**Table 2:** Biomarkers in the TME.

Classification	Biomarkers	Drug	Results	Reference
**Tumour microenvironment biomarkers**	PD-L2	Nivolumab, ipilimumab	Patients with positive PD-L2 expression or low PD-L2 DNA methylation and high PD-L2 mRNA expression achieved higher OS and PFS.	(43, 44)
	HHLA2	Immune-checkpoint inhibitor	Responders had significantly higher HHLA2 expression levels than non-responders. The ORR was 50% in HHLA2-positive patients and 14.3% in negative patients.	(45)
	CD8+ T cells	Pembrolizumab	Patients with positive PD-L1 expression and higher levels of CD8+ TILs had a better prognosis. The increased presence of CD8+ T cells tumor-resident is strongly associated with better melanoma-specific survival.	(46–48)
	BCR	None	Decreased BCR gene segment diversity was associated with improved survival in melanoma.	(49)
	TAM	None	Patients with MM of higher TAM levels usually indicate a poor prognosis.	(50)
	COL3A1	Immune-checkpoint inhibitor	Patients with COL3A1 mutations showed a significantly improved OS and PFS compared with patients without such mutations	(51)
	PTEN	PD-1/PD-L1 inhibitor	Loss of PTEN in MM patients increases tumour resistance to immunotherapy. Patients with MM with PTEN hypermethylation had shorter survival.	(52, 53)
	EGFR	Pembrolizumab, nivolumab	Patients with high baseline EGFR expression had a significantly higher relapse rate than those with low EGFR expression.	(54)
	MDM2	Immune-checkpoint inhibitor	Patients with MDM2/MDM4 amplification developed TTF <2 months and showed a clearly accelerated rate of tumor growth compared to that before treatment.	(55)
	FGFR	Immune-checkpoint inhibitor	Patients with FGFR mutations had better median-OS than those without (60.00 vs. 31.00 months).	(56)
	STK11	Immune-checkpoint inhibitor	Mutations or phenotypic defects in STK11 have been significantly associated with immunotherapy resistance in patients with metastatic melanoma.	(57, 58)
	IFN-γ	Atezolizumab, pembrolizumab	Expression of IFN-γ and IFN-γ-inducible genes was elevated in tumors before treatment in responding patients.	(59, 60)
	JAK1/2, B2M	PD-1 inhibitor	Patients with MM harboring JAK1/2-inactivating and B2M-truncating mutations did not respond to anti-PD-1 drugs.	(61, 62)
	CAF	PD-1 inhibitor	CAF significantly impaired the generation of melanoma-specific T-cell responses and compromise the efficacy of anti-PD-1 checkpoint inhibition.	(63)
	S100A8/A9	Pembrolizumab	Patients with high baseline S100A8/A9 showed significantly impaired survival compared to patients with low baseline S100A8/A9.	(64)

PD-L2, programmed cell death 1 ligand 2; OS, overall survival; PFS, progression-free survival; HHLA2, human endogenous retrovirus-H long repeat-associating 2; ORR, objective response rate; BCR, B-cell receptor; TAM, Tumour-associated macrophage; MM, metastatic melanoma; COL3A1, collagen alpha-1 (III); PTEN, phosphatase and tensin homolog deleted on chromosome 10; MDM2, mouse double minute 2 homolog; TTF, time-to-treatment failure; FGFR, fibroblast growth factor receptor; STK11, serine/threonine kinase 11; IFN-γ, interferon-γ; JAK1/2, Janus kinases 1/2; B2M, β2-microglobulin; CAF, cancer-associated fibroblast.

PD-L2 is another important ligand in PD-1/PD-L1 signalling pathway, which can bind to PD-1 to suppress cellular immunity. Moreover, PD-L2 has stronger affinity than PD-L1. This is because the tryptophan side chain of PD-L2 is exposed to the surface of the protein and can interact with PD-1, while the corresponding structure of PD-L1 is hidden in the hydrophobic core of the protein (65). In a study in 147 patients with metastatic melanoma, expressions of both PD-L1 and PD-L2 correlated significantly with increasing densities of immune cells in the tumour specimens and with immunocytes. Patients with positive PD-L2 expression had significantly prolonged OS regardless of 5% or 20% expression levels. Patients whose tumours were double positive for PD-L1 and PD-L2 had better OS than patients with tumours that were not double positive. PD-L2, alone or in combination with PD-L1, may predict clinical outcomes in patients with MM (43). In an epigenetic study of PD-L2, DNA promoter hypomethylation and high mRNA expression were strong predictors of prolonged OS. Low PD-L2 DNA methylation and high PD-L2 mRNA expression in pre-treatment melanoma samples from anti-PD-1-treated patients predicted longer progression-free survival. PD-L2 DNA methylation and mRNA expression also correlated with TILs and IFN-γ signature. The IFN-γ-induced PD-L2 expression in melanoma cells is controlled by the extent of DNA methylation in the PD-L2 promoter region (44).

HHLA2 is a specific molecule in TME ubiquitously and highly expresses in many cancers and belongs to the B7 family. A study suggested that the OS and PFS was significantly longer in skin cutaneous melanoma (SKCM) patients with higher HHLA2 expression levels. The immune infiltration levels and TME scores were obviously higher in the HHLA2 high group. For immunotherapy patients, responders had significantly higher HHLA2 expression levels than non-responders. The ORR was 50% in HHLA2-positive patients and 14.3% in negative patients (45).

Tumor-infiltrating lymphocytes (TILs) exist in the TME, and its components include cytotoxic T cells, regulatory T cells, B cells, etc. They can directly or indirectly participate in the immune response, affect tumour growth and treatment response. The presence of TILs is critical for immunotherapy, no matter it is TILs treatment that returns to the patient *in vivo* after direct extraction of amplification, or treatment that restores T cell function through immune checkpoint inhibitors; the effect is influenced by the number and functionality of TILs. A combination of PD-L1 and TILs statuses may lead to a more accurate prognosis than a single PD-L1-level status. Tumours have been divided into four distinct subgroups according to PD-L1 and TILs status, and patients with positive PD-L1 expression and high TILs levels are considered to have adaptive immune resistance (66).

The CD8+ T cells play a critical role in tumour control, particularly in melanoma. In a Korean study involving 63 patients with melanoma, patients with positive PD-L1 expression and low levels of CD8+ TILs had a worse prognosis (46). Studies have demonstrated that the higher the density of CD8+ T cells at baseline in metastatic melanoma, the better the prognosis of PD-1 immunotherapy (47). A subset of antigen-experienced CD8+ T cells become resident within tissue environments, facilitated by local cytokines. Tissue-resident CD8+ T cells are characterized by the constitutive expression of CD69 and CD103. A study of advanced stage MM patients being treated with anti–PD-1 monotherapy showed that tumor-resident CD8+ T-cell numbers were more prognostic than total CD8+ T cells in metastatic melanoma. The increased presence of tumor-resident CD8+ T cells was strongly associated with better melanoma-specific survival in untreated patients (48).

The B cells also play an important role in anti-tumor immunity in TME. A genomic analysis of immune cell infiltrates across 11 tumour types showed that high expression of T-cell and B-cell signatures predicted improved OS in melanoma. B-cell gene signature expression was more statistically significantly associated with improved OS than T-cell gene signature expression. Decreased BCR gene segment diversity was associated with improved survival in melanoma (HR = 2.67) (49).

TAM is considered to play an important role in tumour growth, progression and metastasis. Macrophages can be divided into M1 and M2 subtypes according to the cell phenotype. M1 subtype macrophages have anti-cancer effects, while M2 subtype macrophages can promote tumour development. Different subtypes of macrophages influence the prognosis of cancer patients and their response to immunotherapy (50). In melanoma, high-density macrophages are often suggestive of a poor prognosis, which may be related to the fact that melanoma exosomes can stimulate TAM polarization, promoting tumour growth and metastasis (67).

The TILs can also be altered by gene mutations or increased receptor expression in some tumour cells or other cells in TME. COL3A1 gene encodes alpha 1 chain of type III collagen, which is an extracellular matrix protein and a major structural component in hollow organs such as large blood vessels (68). The alterations in COL3A1 have been demonstrated to be associated with melanoma metastasis. A study of 631 melanoma patients who received blockade therapy of immune checkpoints showed that patients with COL3A1 mutations showed a significantly improved OS and PFS compared with patients without such mutations. The COL3A1-mutated tumours exhibited an elevated ORR and disease control rates. CD8+ T cells, activated CD4+ memory T cells, and resting NK cells infiltrated tumours of patients with COL3A1 mutations. Furthermore, the COL3A1 mutant tumours exhibited increased infiltration of pro-inflammatory M1 macrophages and decreased infiltration of immune-suppressive M2 macrophages. The favourable genomic traits and the immune microenvironment may underlie the better immunotherapy response of COL3A1 mutations (51).

The phosphoinositide 3-kinase (PI3K) pathway plays a critical role in cancer by regulating several critical cellular processes, including proliferation and survival. One of the most common ways that this pathway is activated in cancer is by loss of expression of the tumour suppressor PTEN, which is a lipid phosphatase that dampens the activity of PI3K signalling. Loss of PTEN occurs in up to 30% of melanomas. A study of preclinical models demonstrated that loss of PTEN promoted resistance to immunotherapy in melanoma by reducing CD8+ TILs (52). The methylation of the PTEN gene leads to its inactivation. In a review of the methylation levels of the PTEN gene in 158 Korean melanoma patients, it was found that the PTEN hypomethylated subgroup had a relatively longer survival, whereas the PTEN hypomethylated subgroup had a shorter survival, indicating significant difference between them (p = 0.017) (53).

EGFR is a growth factor receptor that induces cell differentiation and proliferation upon activation through the binding of one of its ligands. EGFR activation is associated with upregulation of PD-1, PD-L1 and CTLA-1, which can drive immune escape. EGFR is frequently expressed at elevated levels in different forms of cancer and expression often correlates positively with cancer progression and poor prognosis (69). PD-1 immunotherapy is not recommended for patients with EGFR-mutant cancers, and available data suggest that immunotherapy is ineffective in patients with EGFR mutations and even presents a risk of explosive progression. One study included 137 treatment-naive patients treated with adjuvant pembrolizumab or nivolumab. Patients with high baseline EGFR expression had a significantly higher relapse rate than those with low EGFR expression. Patients with high EGFR expression had significantly worse outcomes, and the median relapse-free survival for these patients was 3 months; for patients with low EGFR expression, the median relapse-free survival was not reached. It identified EGFR expression in therapy-naive metastatic tissue as a potential negative predictive factor for immunotherapy (54).

MDM2 is a negative regulator of the tumour suppressor p53 and is often highly expressed in solid tumours. p53 protein is a tumour suppressor, which plays a role in regulating cell growth cycle. For T cells it was shown that MDM2 inhibition can induce production of tumour necrosis factor α and IFN-γ, whereas in melanoma cells MDM2 inhibition induces interleukin-15 (IL-15) production. IL-15 serves as an activator of anti-tumor CD8+ T cells and natural killer cells. MDM2 amplification is found in about 7% of cancers; it inhibits the p53 tumour suppressor. MDM4 is a homolog of MDM2 that interacts with it and also inhibits p53 (70). A study of 155 patients who received immunotherapy identified that those with melanoma were more likely to develop time-to-treatment failure (TTF) <2 months compared with patients with other tumours. Patients with MDM2/MDM4 amplification were taken off immunotherapy in less than two months, and four showed a clearly accelerated rate of tumour growth compared to that before treatment. In that study, EGFR alterations was also described as a high risk factor. Among 10 patients with EGFR alterations, eight had a TTF <2 months (55).

The FGFR family consists of four highly conserved transmembrane receptors, FGFR1-4, which play key roles in embryonic development, proliferation, angiogenesis, and tumour metastasis. FGFR is mutated across numerous cancers, triggering the FGFR signalling pathway, hence promoting tumour progression (71). Melanoma had the highest mutation frequency of 22.05% among 27 cancers. In the immune checkpoint inhibitors-treated cohort, patients with FGFR mutations had better survival than those without (median OS: 60.00 vs. 31.00 months). The ORR was higher for patients harbouring FGFR mutations (55.56%) compared to wild-type patients (22.40%). FGFR mutant melanoma tended to exhibit an enhanced anti-tumor and inflammatory tumour immune microenvironment, which synergistically promotes inflammatory to activate immune cells to kill cancer cells (56).

The STK11 gene codes for liver kinase B1, a highly conserved serine/threonine kinase, is implicated as a tumour suppressor in epithelial cancer invasion and metastasis. For the STK11-deficient population, the efficacy of immunotherapy was significantly reduced. This may be related to the fact that STK11 deficiency leads to T cell suppression, resulting in reduced numbers of tumour-infiltrating lymphocyte and decreased expression of PD-L1 in tumours (72). Mutations in STK11 have been reported to be significantly associated with immunotherapy resistance in patients with MM (57). But in addition to mutation status, the phenotypic classification of STK11 is also important for survival prognosis. Among the patients whose mutation status was not consistent with the phenotype classification, the immunotherapy efficacy was significantly worse in the patients without STK11 mutation but with defective phenotype than in the patients with STK11 mutation but with normal phenotype (58).

TILs produce cytokines such as IL-2 and IFN-γ, which play an important role in the activation and proliferation of TILs. IFN-γ can induce PD-L1 expression in tumour cells and macrophages, suggesting that IFN-γ may be an important factor in inducing PD-L1 expression in the TME. IFN-γ signalling and the associated biology of T cell cytolytic activity, antigen presentation, and chemokine production are important components of a PD-1 checkpoint blockade–responsive immune microenvironment in melanoma. In a phase I study of atezolizumab for patients with advanced melanoma, elevated expression of IFN-γ as well as IFN-γ-inducible genes (for example, indoleamine 2,3 dioxygenase 1 [IDO1] and C-x-C motif chemokine ligand 9 [CXCL9]) in pre-treatment tumours were demonstrated in responding patients (59). A study analyzed gene expression profiles using RNA from baseline tumour samples of pembrolizumab-treated patients. To examine immune-related gene expression signatures associated with pembrolizumab response in patients with metastatic melanoma. Many of the top-ranked genes were directly linked to IFN-γ signalling and showed correlation with the expression of IFN-γ. A 10-gene “preliminary IFN-γ” signature was constructed that was able to separate responders and nonresponders to pembrolizumab among the 19 pilot data patients with melanoma (60).

IFN-γ through the interferon gamma receptor, the Janus kinases JAK1 and JAK2 and the signal transducers and activators of transcriptions (STATs) results in the expression of a large number of interferon-stimulated genes and lead to beneficial anti-tumor effects. The melanoma cell lines that had JAK1/2 homozygous loss of function mutations did not respond to IFN-γ. β2-microglobulin (B2M) is an important subunit of MHC class I which exerts substantive biological functions in tumourigenesis and immune control. Acquired resistance to PD-1 blockade in patients with advanced melanoma can be associated with loss-of-function mutations with loss of heterozygosity in JAK1/2 or in B2M. Two cancer patients with higher TMB but JAK1/2-inactivating mutations did not respond to PD-1 immunotherapy (62). Sequencing of the genes of four other drug-resistant MM patients revealed that mutations in their JAK1/2 or B2M genes were a major cause of drug resistance (61).

Cancer-associated fibroblasts (CAFs) contribute to the development of a physical barrier that interferes with immune cell infiltration, directly inhibiting T-cell trafficking, and expressing an array of factors, including C-C motif ligand 2 (CCL2), that contribute to the establishment of an immunotolerant TME. A preclinical study suggested that melanoma-associated fibroblasts (MAFs), significantly impair the generation of melanoma-specific T-cell responses and compromise the efficacy of anti-PD-1 checkpoint inhibition (63).

S100A8/A9 is a member of the damage-associated molecular pattern (DAMP) (also known as alarmins) that is released upon cell stress or damage promoting thereby an inflammation. The inflammation-associated S100A8 and S100A9 have been identified to attract melanoma cells and described as a critical factor for recruitment of myeloid-derived suppressor cells (MDSCs) and stimulation of their immunosuppressive functions in the TME (73). In a study of patients with advanced melanoma revealed an exclusive and abundant expression of S100A8/A9 in cells of the TME, mainly granulocytes. High numbers of S100A8/A9 expressing cells in melanomas would translate into elevated S100A8/A9 serum levels. Patients with high baseline S100A8/A9 >5.5 mg/l showed significantly impaired survival compared to patients with low baseline S100A8/A9 in two independent cohorts of patients treated with pembrolizumab (HR 5.37,10.70) (64).

### 4.2 Peripheral circulation biomarkers

Circulating biomarkers are molecules that can be detected in the blood after release, including sugars, cholesterol, proteins, and even intact cells. They can be measured objectively, and some of them can be used as indicators of tumour progression. Circulating biomarkers associated with PD-1/PD-L1 immunotherapy include lactate dehydrogenase (LDH), neutrophil-to-lymphocyte ratio (NLR), soluble PD-L1 (sPD-L1), soluble PD-1 (sPD-1), BCL-2 interacting mediator of cell death (BIM), relative lymphocyte and eosinophil granulocyte counts, and circulating extracellular vesicles (EVs) (**Table 3**).

**Table 3:** Biomarkers in the peripheral circulation.

Classification	Biomarkers	Drug	Results	Reference
**Peripheral circulation** **biomarkers**	LDH	Ipilimumab	Patients with baseline serum LDH concentrations more than 2 times the upper limit of normal and those with elevated serum LDH levels during the first two weeks of immunotherapy showed a significantly shorter OS.	(74, 75)
	NLR	Ipilimumab	Patients with NLR ≥5 showed a lower OS.	(76–78)
	sPD-L1	Ipilimumab, pembrolizumab	Patients with moderate to low baseline serum sPD-L1 levels and those with reduced serum sPD-L1 levels during immunotherapy showed better clinical responses.	(79)
	sPD-1	Nivolumab	Patients with elevated serum sPD-1 levels during immunotherapy showed a better prognosis.	(80)
	BIM	Pembrolizumab	Patients with higher expression of BIM protein on CD8+ T cells in peripheral blood responded better to immunotherapy.	(81)
	Relative lymphocyte and eosinophil granulocyte counts	Nivolumab, pembrolizumab	Higher levels of lymphocyte and eosinophil granulocyte at baseline are associated with better outcomes. An increase in the number of absolute eosinophil granulocyte and a decrease in the number of relative lymphocyte showed a worse prognosis after immunotherapy.	(82, 83)
	Circulating EVs	Nivolumab, pembrolizumab	Patients with higher levels of circulating EVs had a poor prognosis.	(84)

LDH, lactate dehydrogenase; NLR, neutrophil-to-lymphocyte ratio; sPD-L1, soluble PD-L1; sPD-1, soluble PD-1; BIM, BCL-2 interacting mediator of cell death; EVs, extracellular vesicles.

LDH is a kind of NAD-dependent kinases, which can catalyze the oxidation reaction between pyruvate and lactic acid. During the growth, invasion and metastasis of tumour cells, serum LDH level may increase. LDH can be a good predictive biomarker for patients with malignant melanoma. Evidence have shown that patients with higher-than-normal serum LDH levels at baseline generally have a worse prognosis and that patients with higher-than-normal serum LDH levels at 2 times the upper limit are less likely to benefit in the long term from ipilimumab treatment (74). Patients with elevated serum LDH levels during the first two weeks of PD-1 immunotherapy had shorter survival (75). The rapid growth and proliferation of tumour cells may result in impairment of surrounding normal tissues, which can also be destroyed during their invasion and metastasis, allowing LDH to be released into the blood. Fast-growing tumour tissue usually gets its energy from glycolysis and also produces a lot of LDH. Elevated levels of LDH in the blood can lead to a decrease in the pH of the TME, which negatively affects the function of the lymphocyte and the efficacy of the immune response (85).

The higher the baseline NLR level in cancer patients, the worse the prognosis. An elevated NLR ratio usually indicates increased neutrophil, reflecting a state of systemic inflammation. The inflammatory reaction can promote the proliferation and survival of tumour cells, as well as the formation and metastasis of blood vessels. In particular, increased neutrophil can suppress adaptive immune responses in the TME, while decreased lymphocyte counts indicate a state of immunosuppression (86). Studies have shown greater improvement in PFS and OS with ipilimumab in melanoma patients with baseline NLR <5 than patients with baseline NLR ≥5 (76). The NLR of 5 was considered a cutoff point, and NLR ≥5 was an independent prognostic risk indicator in the multivariate analysis model, indicating poor OS (77). In a retrospective study, which was more focused than ever on the analysis of the association of pre-treatment peripheral blood NLR with survival and response rates in patients treated with immune checkpoint inhibitors, the distribution of NLR in different cancer species was investigated, which was consistent with the predictive value. Using the NLR as an independent cohort predictor, high NLR was observed to be associated with significantly worse OS as well as lower response rates (17% vs. 28%) and clinical benefit (26% vs. 41%) (78).

Soluble PD-L1 is associated with a poor prognosis in many tumours, which may be related to its production mechanism. The serum sPD-L1 of the patient has multiple sources of production, including the intrinsic splicing of secretory cells, pre-tumour inflammatory response and anti-tumour immune response. Several melanoma cell lines have been found to secrete PD-L1 splice variants lacking transmembrane structure, namely sPD-L1 variants. It can inhibit T cell activation and proliferation. The higher circulating sPD-L1 levels at tumour baseline may represent the greater tumour burden of the patient, increased aberrant splicing activity in tumour cells, or decreased anti-tumour immunity, leading to a poor response to immunotherapy. However, response to immune checkpoint inhibitors was the highest in patients with a moderate increase in sPD-L1 at baseline (79). Similar to sPD-L1, sPD-1 is a shear variant formed by the removal of the transmembrane structure by the full-length PD-1. Soluble PD-1 can block PD-1/PD-L1 pathway by binding membrane PD-L1 competitively, enhance T cell immunity and exert its anti-tumour effect (87). The presence of progressive or stable disease was associated with increased sPD-L1 when nivolumab was used to treat renal cell carcinoma (RCC) and melanoma. In patients in remission, circulating sPD-L1 levels were stable or decreased (88). For patients with advanced melanoma, incremental levels of serum sPD-1 during treatment were a good biomarker for the combination of nivolumab and ipilimumab, but it was not associated with the treatment effect of pembrolizumab (80).

The BIM gene (BCL2L11 gene) is responsible for encoding the BIM protein in the BCL2 protein family, whose function is to induce apoptosis by binding to anti-apoptotic proteins of other BCL2 families through a highly conserved BH3 domain. Melanoma is known to upregulate the BIM protein level of anti-cancer T cells through PD-1/PD-L1 signalling pathway, inducing T cells apoptosis and achieving the goal of immune evasion. Because BIM levels at tumour baseline can reflect the intensity of the PD-1/PD-L1 signalling pathway in tumour patients, and the method of detecting BIM levels is simpler than the ICH method for assessing PD-L1 expression, T-cell BIM level is expected to be a predictive biomarker. Studies have demonstrated higher levels of BIM in CD8+ T cells in the peripheral blood of patients who respond to anti-PD-1 therapy (81). In addition, repeated assessment of T-cell BIM levels during treatment provides greater flexibility in determining drug efficacy and discontinuation time. In general, a decrease in BIM levels on treatment predicts a desirable response to immune checkpoint inhibitors in patients, as this implies that immunotherapy may have successfully blocked the PD-1/PD-L1 pathway. If BIM levels remain or increase during treatment, this may represent resistance or a false progression (89).

Circulating complete blood count is an objective method that allows us to visualize the blood changes of cancer patients before and after immunotherapy. Its role in tumour prognosis has been a hot topic in clinical research. Among them, eosinophil granulocyte count and lymphocyte count have been reported to have prognostic significance in many literatures. After the initiation of immunotherapy, relative lymphocyte counts decreased from baseline in nivolumab-treated melanoma patients if their absolute eosinophil granulocyte counts increased by more than 3.2%, the probability of poor prognosis increased (82). There is also evidence that higher lymphocyte and relative eosinophil granulocyte counts at baseline are associated with benefit from pembrolizumab for patients with melanoma (83). This may be related to the CD28-B7 interaction that induces allergic inflammation leading to an increase in the number of eosinophil granulocyte (90).

Ideally, detection of circulating markers could serve as an objectively effective strategy to meet the need for predicting treatment outcomes and monitoring disease progression in real time. Much attention is focused on normal and cancer cell-released circulating EVs in the hope that it can be used clinically to evaluate cancer patients. EVs are membrane-bound spheres released by cells and carry lipids, soluble and transmembrane proteins, and various RNA species, including mRNA, miRNA and DNA. Cancer cell-derived EVs are emerging as local and systemic intercellular mediators of oncogenic information (91). A study of 71 MM patients showed that higher levels of circulating EVs predicted worse survival (PFS, OS, and ORR) in patients with MM, and high levels of PD-1 EVs in CD8+ T cells and PD-L1 EVs in melanoma cells were independent biomarkers for predicting PFS. This study demonstrated that circulating EVs reduced the immune capacity of peripheral blood mononuclear cells (PBMCs), suggesting that the therapeutic efficacy of PD-1/PD-L1 mabs can be enhanced by eliminating circulating EVs to reactivate patient PBMC (84).

### 4.3 Gene mutations

Tumour mutation burden (TMB) is generally defined as the number or proportion of mutations occurring in a tumour sample, usually expressed as the number of mutations per megabase. Its numerical value can reflect the potential of tumour to produce neoantigens, and it has been proved to be able to predict the efficacy of immunotherapy for a variety of tumours. Now, high TMB (TMB-H, TMB ≥10 mut/Mb) has been approved by the FDA as a diagnostic marker for disease progression after prior treatment with pembrolizumab, deficiency of satisfactory alternative treatment options, and unresectable or metastatic solid tumours in children and adults. A study performed a multiomic profiling of baseline tumours in 77 patients with advanced cutaneous melanoma treated with anti-PD-1 with or without anti-CTLA-4 showing that overall response to any treatment was significantly associated with higher TMB (92). Tumours with a high TMB and a high IFN-γ signature show the best response to immunotherapy. In general, TMB refers to tissue TMB (tTMB), but with the development of liquid biopsy techniques, for patients with difficulty in obtaining tissue specimens, their immunotherapy efficacy and dynamically monitor treatment changes can be predicted by evaluating blood TMB (bTMB). One study showed that bTMB was significantly associated with tTMB and was able to substitute TMB for prognosis in cancer patients (93).

TMB is usually not tested alone, but often in conjunction with DNA mismatch repair (MMR) and microsatellite instability (MSI). MMR can accurately identify and repair base mismatches, small base deletions and insertions during DNA replication and recombination, playing an important role in maintaining genome stability. MSI is a microsatellite sequence length change caused by insertion or deletion mutations during DNA repair. In general, patients with high MSI (MSI-H) and those who appear deficient MMR (dMMR) are more likely to be recognized by the immune system, and the prognosis of immunotherapy is better. This is because MSI-H and dMMR tumours harbor a large number of mutations and potential neoantigens, whereas host T cells do not acquire immune tolerance for these neoantigens and are therefore more likely to be recognized by the immune system. In a proof-of-concept study of patients with advanced dMMR cancer in 12 different tumour types, PD-1 immunotherapy delivered desirable responses, with an ORR of up to 53% (94).

In addition to TMB, which reflects the overall mutational level of cancer patients, many individual gene mutations are also strongly associated with the efficacy of PD-1/PD-L1 immunotherapy. According to its effect on immunotherapy, it can be divided into sensitive gene and inefficient gene (**Table 4**).

**Table 4:** Gene mutation as a biomarker.

Classification	Biomarkers	Drug	Results	Reference
**Sensitive gene mutations**	COL3A1	Immune-checkpoint inhibitor	Patients with COL3A1 mutations showed a significantly improved OS and PFS as compared with patients without such mutations	(61)
	FGFR	Immune-checkpoint inhibitor	Patients with FGFR mutations had better median-OS than those without (60.00 vs. 31.00 months).	(56)
	BRCA	None	Higher PD-1 expression on CD8+ T cells in BRCA1/2 mutant tumours may be beneficial in enhancing the effects of immune checkpoint inhibitors.	(95)
	ATM	None	Tumours with ATM mutations responded significantly better to immunotherapy.	(96)
	POLE/POLD1	Immune-checkpoint inhibitor	Patients with POLE or POLD1 mutations had significantly longer OS at 34 months compared with 18 months in the wild-type population.	(97)
	NOTCH	PD-1/PD-L1 inhibitor	Patients with tumours harboring NOTCH mutations benefit more from immunotherapy, demonstrating longer PFS and OS.	(98, 99)
	KMT2C	PD-1 inhibitor	Patients harboring KMT2C mutations showed significantly better OS as compared with wild-type KMT2C.	(100)
**Inefficient gene mutations**	PTEN	PD-1/PD-L1 inhibitor	Loss of PTEN in MM patients increased tumour resistance to immunotherapy. Patients with MM with PTEN hypermethylation had shorter survival.	(52, 53)
	EGFR	Pembrolizumab, nivolumab	Patients with high baseline EGFR expression had a significantly higher relapse rate than those with low EGFR expression.	(54)
	MDM2/4	Immune-checkpoint inhibitor	Patients with MDM2/4 amplification developed TTF <2 months and showed a clearly accelerated rate of tumor growth compared to that before treatment.	(55)
	STK11	Immune-checkpoint inhibitor	Mutations or phenotypic defects in STK11 have been significantly associated with immunotherapy resistance in patients with metastatic melanoma.	(57, 58)
	JAK1/2, B2M	PD-1 inhibitor	Patients with MM harboring JAK1/2-inactivating and B2M-truncating mutations did not respond to anti-PD-1 drugs.	(61, 62)
	PBRM1	PD-L1 inhibitor	The trends of PBRM1-mutant patients towards a worse survival from immunotherapy.	(101)
	TERT	None	TERT-mutated melanoma patients had a significantly worse OS compared to wild-type ones.	(102)
	ALK	None	PD-1 immunotherapy is not recommended for ALK mutation-positive patients.	(103)
	BRAF V600E/K	Pembrolizumab	The prognosis of MM patients with BRAF mutation had a lower ORR compared with patients with BRAF wild-type.	(104)
	MDM2, EGFR	PD-1/PD-L1 inhibitor	Some patients with MDM2 family amplification or EGFR aberrations had hyper-progression after immune checkpoint inhibitors-treat.	(55)

COL3A1, collagen alpha-1(III) gene; FGFR, fibroblast growth factor receptor; BRCA, breast cancer susceptibility gene; ATM, ataxia telangiectasia-mutated gene; POLE, polymerase epsilon; POLD, DNA polymerase delta; PTEN, phosphatase and tensin homolog deleted on chromosome 10; EGFR, epidermal growth factor receptor; MDM2, mouse double minute 2 homolog; STK11, serine/threonine kinase 11; JAK, Janus kinase; B2M, β2-microglobulin; PBRM1, polybromo 1; TERT, telomerase reverse transcriptase; ALK, anaplastic lymphoma kinase; BRAF, v-raf murine sarcoma viral oncogene homolog B1.

PD-1 immunotherapy can achieved desirable response in cancer patients with sensitive gene mutations, including COL3A1, FGFR, breast cancer susceptibility (BRCA), ataxia telangiectasia-mutated gene (ATM), polymerase epsilon (POLE), DNA polymerase delta 1 (POLD1), NOTCH, KMT2C genes and so on. The previously mentioned MSI and MMR also fall into this range.

BRCA is a group of genes directly related to hereditary breast cancer, including BRCA1 and BRCA2. Under normal physiological conditions, the BRCA1/2 gene is tumour suppressor gene and plays an important role in regulating the replication of human cells, the repair of DNA damage and the normal growth of cells. When BRCA1/2 is mutated, it can increase the risk of many cancers. CD8+ T cells have higher PD-1 expression in BRCA1/2 mutant tumours, which may enhance susceptibility to PD-1/PD-L1-targeted immune checkpoint therapy (95).

ATM protein plays a central role in sensing DNA double-strand breaks and coordinating their repair. When the ATM gene is deleted, it can promote the efficacy of immunotherapy through promoting cytoplasmic leakage of mitochondrial DNA and activation of the cyclic GMP-AMP (cGAS) and stimulator of interferon genes (STING) pathway (96). But more clinical data are needed to confirm the effect.

POLE and POLD1 genes encode the catalytic subunits of DNA polymerase ϵ and δ, respectively, which play an important role in DNA replication and correction, and their mutations directly affect the occurrence and development of tumours. An analysis of a database of 47721 patients with different types of cancer found that patients with POLE/POLD1 mutations nearly doubled their OS after immunotherapy as compared with those with wild-type disease (97).

Notch pathway has been shown to play an important role in the proliferation, differentiation, apoptosis and invasion of a variety of tumour cells. In addition, increasing researches confirms that NOTCH mutations are associated with a favourable prognosis for immunotherapy. A combined analysis of cancer patient information from multiple open databases found that NOTCH mutations increased the efficacy of PD-1 inhibitors by 2.2-fold and reduced the risk of disease progression by 39%; patients had a 44% lower risk of death (98). Neuroblastoma rat sarcoma (NRAS) gene is one of the most significant driver genes in melanoma. The NRAS wildtype patients account for 80%-85% of melanomas and have a better prognosis. For patients with NRAS wild-type MM, NOTCH4 mutations have better PFS and OS (99).

KMT2C is a well-known epigenetic regulator that plays an important role in transcriptional regulation, specifically activation, by loosening chromatin structure through its catalytic role in mono-methylation of histone H3 lysine K4 (H3K4). KMT2C was shown to contribute to the genomic stability and its mutation leads to the obvious TMB elevation in multiple cancers. In a study based on three published MM cohorts, MM patients harboring KMT2C mutations showed significantly better OS after treatment with PD-1 monoclonal antibody as compared with wild-type KMT2C. These results indicate that KMT2C might be a reliable independent biomarker in response to anti-PD-1 treatment of MM patients (100).

PD-1 immunotherapy is ineffective for patients with inefficient gene mutations. The main mutations include PTEN, EGFR, STK11, JAK1/2, B2M, polybromo 1 (PBRM1), telomerase reverse transcriptase (TERT), anaplastic lymphoma kinase (ALK), BRAF, MDM2 genes and so on. Part of those has been described above.

PBRM1 encodes a subunit of the SWI/SNF chromatin remodeling complex (PBAF subtype), which is involved in cell differentiation, proliferation and DNA repair. Approximately 80% of PBRM1 somatic mutations may result in loss of function of the protein. A comprehensive analysis of PBRM1 in multiple cancer types showed that the frequency of PBRM1 mutations was 8.4% in SKCM. Truncating mutations were the most common type of mutation. Patients with PBRM1 mutations showed a shorter median OS. The trends of PBRM1-mutant patients towards a worse survival and high TMB towards clinical benefit from immunotherapy were observed (101).

TERT mutations allow tumour cells to grow without restriction and are found in many types of tumours. In a comprehensive literature review and meta-analysis, data from 19 independent studies showed that TERT-mutated melanoma patients had a significantly worse OS as compared with wild-type ones. TERT mutations adversely affected the survival of patients with melanoma (102).

ALK is an oncogene responsible for the production of the protein receptor tyrosine kinase. ALK expression has been identified in metastatic and primary melanomas. ALK expression in melanoma resulted from a novel transcript variant, known as ALK^ATI^. ALK^ATI^, similar to ALK, may be sensitive to targeted therapy. PD-1 immunotherapy is not recommended for ALK mutation-positive patients (103).

In addition, BRAF gene mutation is a common mutation in MM, and it is also an important factor leading to the occurrence and development of MM. Compared with wild type, BRAF mutant MM has a worse prognosis. For patients with MM who received pembrolizumab, patients with a BRAF V600E/K-mutation had a lower ORR as compared with patients with BRAF wild-type, but did not present a significant difference (34.3% vs. 39.8%) (104). The BRAF-targeted therapy or combined with immunotherapy is recommended for patients with BRAF-mutated melanoma.

Shumei Kato et al. described a class of cancer patients who developed abnormal hyper-progressors after immunotherapy. These patients who experienced explosive progression showed TTF <2 months, disease progression on the first CT scan after treatment with the PD-1 antibody, a 50% increase in tumour size, and a more than twofold increase in tumour growth. This study demonstrates that MDM2 family amplification or EGFR aberrations may be associated with accelerated progression (55). The possibility of hyper-progression is very important for patients with the disease, and treatment strategies should be carefully formulated.

## 5 Discussion and perspective

It has been 9 years since the first PD-1/PD-L1 immunotherapy was approved for the market. With the development of the research, the PD-1/PD-L1 immunotherapy has gradually been applied to many kinds of cancers, such as MM, NSCLC, lymphoma. Both monotherapy and combination therapy showed relatively high response rate and low adverse reaction rate, which is undoubtedly a boon to cancer patients. With the expansion of the scale of PD-1/PD-L1 immunotherapy, more problems have been discovered and researched, such as how to improve the response rate, the prediction of efficacy, the mechanism of drug resistance and coping strategies.

CDx can help screen specific patient populations that may benefit from the treatment product, better guiding medication use and reducing health care expenditures. The FDA-approved CDx method for PD-1/PD-L1 immunotherapy is mainly PD-L1 detection by IHC. The IHC method for PD-L1 detection has many limitations, and antibodies, staining thresholds, sampling sites, and subjective judgment of the pathologist can all make the results different. Detection of PD-L1 using molecular diagnostic techniques may help physicians diagnose cancer patients more quickly and accurately. In addition, substantial evidence suggests that using PD-L1 expression status alone to predict patient efficacy is sometimes not accurate.

There is no standard FDA-approved CDx for PD-1/PD-L1 therapy in melanoma. The discovery of more comprehensive predictive new biomarkers is essential for the PD-1/PD-L1 immunotherapy of melanoma and the further development of this field into precision medicine. This article lists the biomarkers of TME, peripheral circulation and gene mutations, covering the main current research directions.

In recent years, increasing attention has been paid to the role of tumour microenvironment in tumour growth, metastatic spread and response to treatment. Understanding the complex interplay between tumour cell-intrinsic, cell-extrinsic, and systemic mediators of disease progression is essential for the rational development of effective anticancer therapies. Many components of TME can affect and help predict the response to PD-1/PD-L1 immunotherapy. PD-1/PD-L1 immunotherapy relies on the patient’s own immune system, especially TILs. “Hot” (immune-inflamed) tumours are more likely to benefit from immunotherapy, whereas “cold” (immune-desert/immune-excluded) tumours are the opposite. Melanoma is usually considered as a “hot” tumour, but some specific gene mutations such as COL3A1, EGFR, etc. can lead to decreased immune infiltration or increased immune checkpoints in TME. The evaluation of the immune status of TME has changed from TILs counting to the detection of TILs quantitative genomic signatures, which is more comprehensive and accurate.

Some components of the TME will enter the peripheral circulation, and the development of tumours will lead to changes in the components in the peripheral circulation. The detection of peripheral blood components is the most convenient and clinically popular, and it can predict treatment outcome and monitor disease progression in real time. The conventional test items LDH and NLR were found to have certain prognostic value of immunotherapy. Soluble PD-1 and circulating EVs, directly related to tumours, are more closely related to tumour status and prognosis.

Independent of PD-L1 and TME, the TMB level of the tumour itself affects its antigenicity, which is also very important for PD-1/PD-L1 immunotherapy. The FDA approval of pembrolizumab for patients with unresectable or metastatic TMB-H or MSI-H or dMMR solid tumours speaks volumes about the prognostic importance of these biomarkers for immunotherapy. Some gene mutations, such as BRCA, ATM, and POLE/POLD1, are not conducive to the prognosis of tumour diseases but are beneficial to immunotherapy. Mutations in other genes, such as PBRM1 and MDM2, can lead to poor prognosis regardless of immunotherapy. Tumours with EGFR, ALK, BRAF mutations may be resistant to PD-1/PD-L1 immunotherapy, and are more suitable for targeted therapy or combination therapy. Although further studies are needed regarding the markers and causes of hyper-progression, genomic testing in patients scheduled for PD-1/PD-L1 therapy may be necessary to determine if their tumours harbor specific alterations associated with hyper-progression.

Patient biomarkers should be tested and monitored before and during treatment, which can not only to guide drug use in real time and predict efficacy, but also to prevent over-treatment and reduce the adverse reactions of patients. Comprehensive assessment using multiple biomarkers may be more effective at predicting response than individual biomarkers. Moreover, the blockade of some biomarkers or related pathways may increase the efficacy of immunotherapy, and the development of new drugs and combination therapies for these biomarkers may be a new therapeutic direction.

## Glossary

ALK: Anaplastic lymphoma kinase
ALK: Anaplastic lymphoma kinase
APC: Antigen-presenting cells
ATM: Ataxia telangiectasia-mutated gene
B2M: β2-microglobulin
BCR: B-cell receptor
BIM: BCL-2 interacting mediator of cell death
BRAF: v-raf murine sarcoma viral oncogene homolog B1
BRCA: Breast cancer susceptibility gene
bTMB: blood TMB
CAF: Cancer-associated fibroblast
CBC: Complete blood count
CCL2: C-C motif ligand 2
CDx: Companion diagnostic
cGAS: cyclic GMP-AMP
COL3A1: Collagen alpha-1(III) gene
CR: Complete response
CTLA-4: Cytotoxic T-lymphocyte-associated protein 4
CXCL9: C-x-C motif chemokine ligand 9
DALYs: Deaths and disability-adjusted life years
DAMP: Damage-associated molecular pattern
dMMR: deficient MMR
EGFR: Epidermal growth factor receptor
ESCC: Esophageal squamous cell carcinoma
EVs: Extracellular vesicles
FDA: Food and Drug Administration
FGFR: Fibroblast growth factor receptor
H3K4: Histone H3 lysine K4
HHLA2: Human endogenous retrovirus-H long repeat-associating 2
HNSCC: Head and neck squamous cell carcinoma
HRD: Homologous recombination deficiency
IC: Immune cells
IDO1: Indoleamine 2,3 dioxygenase 1
IFN-γ: Interferon-γ
IHC: Immunohistochemistry
IL-15: Interleukin-15
JAK: Janus kinases
KRAS: Kirsten rat sarcoma viral oncogene homologue
LDH: Lactate dehydrogenase
LKB1: Liver kinase B1
MAF: Melanoma-associated fibroblast
MDM2: Mouse double minute 2 homolog
MDSC: Myeloid-derived suppressor cell
MHC: Major histocompatibility complex
MIS: Microsatellite instability
MM: Metastatic melanoma
MRR: DNA mismatch repair
MSI-H: high MSI
MST: Median survival time
NCDs: Non-communicable diseases
NLR: Neutrophil-to-lymphocyte ratio
NRAS: Neuroblastoma rat sarcoma
NSCLC: Non-small-cell lung carcinoma
ORR: Objective response rate
OS: Overall survival
PBMC: Peripheral blood mononuclear cell
PBRM1: Polybromo 1
PD-L2: Programmed cell death 1 ligand 2
PFS: Progression-free survival
PI3K: Phosphoinositide 3-kinase
POLD: DNA polymerase delta
POLE: Polymerase epsilon
PR: Partial response
PTEN: Phosphatase and tensin homolog deleted on chromosome 10
RCC: Renal cell carcinoma
SD: Stable disease
SKCM: Skin cutaneous melanoma
sPD-1: soluble PD-1
sPD-L1: soluble PD-L1
STATs: Signal transducers and activators of transcriptions
STING: Stimulator of interferon genes
STK11: Serine/threonine kinase 11
TAM: Tumour-associated macrophage
TC: Tumour cells
TCR: T-cell receptor
TERT: Telomerase reverse transcriptase
TILs: Tumor-infiltrating lymphocytes
TMB: Tumour mutation burden
TMB: Tumour mutational burden
TMB-H: high TMB
TME: Tumour microenvironment
TNBC: Triple-negative breast cancer
TPS: Tumour proportion score
TTF: Time-to-treatment failure
tTMB: tissue TMB
WHO: World Health Organization.
